# Transvaginal Ultrasound Diagnosis of Ovarian Ectopic Pregnancy

**DOI:** 10.7759/cureus.33536

**Published:** 2023-01-09

**Authors:** Marcos Sosa, Sophia Brancazio, Audrey Drummey, Thinh Nguyen, Thomas Toussaint

**Affiliations:** 1 Department of Obstetrics and Gynecology, Lakeland Regional Health, Lakeland, USA; 2 Department of Obstetrics and Gynecology, Carolinas Medical Center, Charlotte, USA; 3 Department of Obstetrics and Gynecology, University of Maryland Shore Women's Health, Easton, USA; 4 Department of Maternal Fetal Medicine, Lakeland Regional Health, Lakeland, USA

**Keywords:** transvaginal sonography, sonography in the diagnosis of ectopic pregnancy, acute pelvic pain, ovarian ectopic pregnancy management, ectopic pregnancy, ovarian cyst

## Abstract

Primary ovarian pregnancies are rare and comprise less than one percent of all ectopic pregnancies. Diagnosis can be difficult as an ovarian ectopic pregnancy may share similar features on ultrasound with those of a corpus luteal cyst. Findings on transvaginal ultrasound, including a hyperechoic ring, may denote the presence of a gestational sac and therefore an ovarian ectopic pregnancy. Ultrasonographic findings, as well as a strong suspicion of an ovarian ectopic pregnancy, are critical. The report reviews the case of a 23-year-old primigravida with first trimester bleeding, an elevated human chorionic gonadotropin, an ovarian cyst, and no intrauterine pregnancy detected on ultrasound. The evaluation, diagnosis, and surgical management of an ovarian ectopic pregnancy are discussed.

## Introduction

Primary ovarian pregnancies are rare and comprise only one percent of all ectopic pregnancies. Diagnosis can be difficult; therefore, ultrasonographic findings and high suspicion are paramount. The diagnosis of an ectopic pregnancy may be made with the assistance of a beta-human chorionic gonadotropin (β-hCG) level. A β-hCG quantitative above a threshold for identification of pregnancy on ultrasound is required and is usually 1,500 mIU/mL [1]. However, the value may be different from institution to institution. Traditional ultrasonographic findings include a gestational sac, a gestational sac with a yolk sac, a fetal pole, or a fetal pole with cardiac activity found in the adnexal region. An ovarian ectopic pregnancy may have the findings noted above, similar to a fallopian tube pregnancy, with the location of the pregnancy being on an ovary. However, the identification of an ovarian ectopic pregnancy may be difficult due to its rarity; therefore, the diagnosis may be less often considered. Another concern is that corpus luteal cysts are common in pregnancy, and an ectopic pregnancy on the ovary may be misidentified as a normal cyst. A "ring of fire" sign seen on ultrasound doppler flow can accompany an ectopic pregnancy in the fallopian tube and is a reference to peripheral blood flow to the gestational sac. However, a "ring of fire" is not highly sensitive for an ovarian ectopic pregnancy, as a corpus luteal cyst on an ovary may have similar findings [2-4].

Primary ovarian pregnancies should be suspected when there is a clinical concern for ectopic pregnancy and no evidence of a fallopian tube pregnancy. Ovarian pregnancies can be misinterpreted as a corpus luteal cyst. Though similarities exist on ultrasound, an internal anechoic area and an echogenic ring may lead to a proper diagnosis and expedite treatment for these patients.

We review the case of a 23-year-old primigravida with first trimester bleeding, an elevated β-hCG, an ovarian cyst, and no intrauterine pregnancy detected on ultrasound. A diagnosis of an ovarian ectopic pregnancy was subsequently made after two laparoscopies. In retrospect, the transvaginal ultrasound findings of an echogenic ring within the ovarian cyst may have led to the immediate diagnosis.

## Case presentation

**Figure 1:**
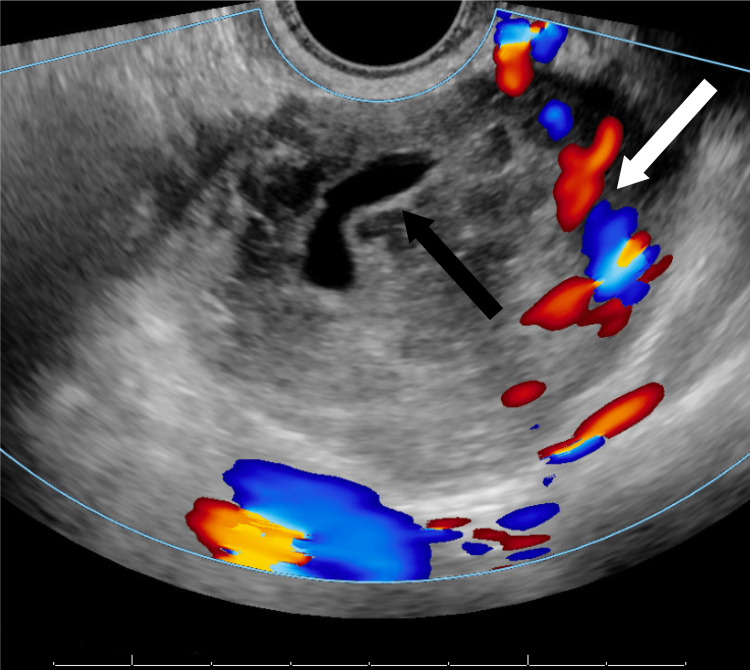
A transvaginal ultrasound depicting an ovarian ectopic pregnancy. The black arrow demonstrates a hyperechoic ring. The white arrow demonstrates a "ring of fire."

A 23-year-old primigravida presented to the emergency department at approximately six weeks' gestation after her last menstrual period with vaginal bleeding. The patient denied any abnormal gynecologic history, including sexually transmitted infections, pelvic pain, or irregular bleeding. She was not using contraceptives because she was actively trying to conceive. The patient's heart rate was 86 beats per minute and her blood pressure was 116/68 mmHg on initial evaluation. Her vitals remained stable during the emergency room visit. She had no abdominal pain on presentation. A pelvic exam revealed a closed cervical os with minimal blood in the vaginal vault. The β-hCG quantitative level was 26,704 mIU/mL, and hemoglobin was 10.0 g/dL. A transvaginal ultrasound revealed no evidence of intrauterine gestation. The ultrasound noted that there was no free fluid in the pelvis. There was no evidence of a mass in the region between the ovary and uterus bilaterally. The left ovary appeared normal. There was a heterogeneous, complex cyst on the right ovary with arterial and venous flow that measured 4.6 x 3.2 x 2.9 cm (Figure 1).

No fetal pole or yolk sac was noted in this area. Due to concerns for ectopic pregnancy and markedly elevated β-hCG, a diagnostic laparoscopy was performed. Intraoperative findings were notable for normal-appearing bilateral fallopian tubes. The right adnexa had a 5 cm ovarian cyst adherent to the right pelvic sidewall. This was thought to represent a corpus luteal cyst, and a cystectomy was not performed. A decision was then made to perform a dilation and curettage to assess for the presence of products of conception. Given the timing of the procedure, a frozen section was unable to be obtained intraoperatively. Given the findings at laparoscopy, the differential diagnosis included a complete abortion versus an ectopic pregnancy with an unidentified location. The patient had stable vital signs, and her hemoglobin level remained stable immediately postoperatively. The patient agreed to return to the clinic in two days for a β-hCG quantitative analysis. The patient recovered well and was discharged home. Pathology results from the dilation and curettage the following day noted fragments of functional polyps and focal stromal decidualization. No products of conception were identified.

**Figure 2:**
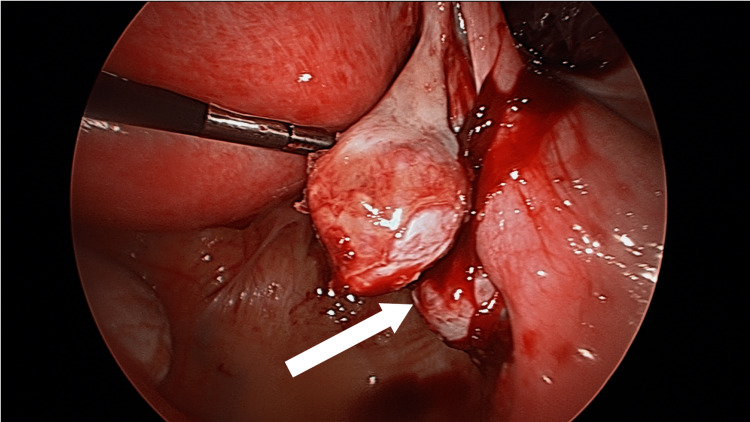
A laparoscopic image of an ovarian ectopic pregnancy. The white arrow demonstrates an ectopic pregnancy attached to the right ovary.

Upon follow-up two days later, the patient had new onset pelvic pain with guarding on physical exam. A repeat ultrasound showed a persistent right ovarian cyst and her repeat β-hCG was 13,259 mIU/mL. Ultrasound findings now included free fluid in the pelvis, which was concerning for a hemoperitoneum. The patient was thus taken back to the operating room for diagnostic laparoscopy and a right ovarian cystectomy. At the time of surgery, there was found to be a right adnexal mass that was adherent to the ovarian fossa (Figure 2).

 

Part of the ovarian cortex was incised, and the tissue was sent for a frozen section to pathology. It was found to be consistent with an ovarian ectopic pregnancy. An ovarian cystectomy was attempted. However, given persistent bleeding despite cauterization and the placement of hemostatic agents, a right oophorectomy was performed. The patient recovered well. On postoperative day one, her quantitative β-hCG appropriately declined to 4,590 mIU/mL. She was discharged home and had an uneventful recovery. Her final pathology report from the ovarian tissue excised resulted in chorionic villi, extensive hemorrhage, and inflammation consistent with an ovarian ectopic pregnancy. Her quantitative β-hCG at her two-week postoperative visit was 0 mIU/mL.

## Discussion

This case highlights the presentation of a rare form of an ectopic pregnancy, the ovarian ectopic pregnancy. Risk factors include those similar to most ectopic pregnancies: history of gonorrhea or chlamydia, history of endometriosis, intrauterine contraceptive device (IUD) use, history of pelvic inflammatory disease (PID), and assisted reproductive technologies (ART) [1-4]. Furthermore, the diagnosis is becoming more common given advancements in ultrasound quality, laparoscopy availability, and an increased prevalence of risk factors [2]. It is important to consider primary ovarian ectopic pregnancies in the setting of an adnexal mass in early pregnancy with no evidence of an intrauterine pregnancy and normal-appearing fallopian tubes in order to facilitate care for these patients. However, its presentation is similar to that of other ectopic pregnancies, and it can often be confused for a ruptured tubal ectopic pregnancy, a ruptured corpus luteal cyst, or even an endometrioma [3].

Four criteria are often used for the intraoperative diagnosis of ovarian ectopic pregnancy. The criteria include a gestational sac located on or in the ovary, an ectopic pregnancy adherent to the ovarian ligament, ovarian tissue noted attached to the wall of the gestational sac on histopathologic evaluation and the fallopian tube on the same side without evidence of ectopic pregnancy [2,5,6]. Transvaginal ultrasound can also serve as a critical tool in diagnosing an ovarian ectopic pregnancy and distinguishing it from other adnexal masses [2,7,8]. First, the presence of a hyperechoic ring in the outer half to one-third of the ovary, denoting the edge of the gestational sac, should raise suspicion for an ovarian ectopic pregnancy and has been noted in the literature [5,7,8]. More attention should be placed on studying the importance of the echogenic ring when evaluating an ovarian ectopic pregnancy. Further studies are needed to assess the sensitivity of using an echogenic ring found on transvaginal ultrasound in order to diagnose an ovarian ectopic pregnancy. In the case presented above, the hyperechoic ring was seen in both transvaginal ultrasounds performed on the patient. The misleading detail was that the gestational sac had collapsed and was not the typical oval or circle shape. A yolk sac or fetal pole was also absent on both ultrasounds, which led to the radiologic evaluation being more complicated. A fetal pole or yolk sac seen on transvaginal ultrasound certainly denotes a pregnancy in the ovary; however, in some instances, neither is present, as in this case. Second, doppler studies may note blood flow around the periphery of an ovarian ectopic pregnancy, the "ring of fire" sign; however, these can also be seen at the periphery of a corpus luteal cyst [4]. Further studies on the use of doppler flow are needed with regard to ovarian ectopic pregnancies. 

Treatment includes both medical and surgical options. Methotrexate can be considered depending on the β-hCG level and findings on ultrasound. Medical management with methotrexate may vary based on criteria from institution to institution. Ovarian preservation with the excision of the ectopic pregnancy can be attempted via ovarian wedge resection or cystectomy, though not always successful given the hypervascularity associated with ovarian ectopic pregnancies. Thus, prior to performing an oophorectomy on a reproductive-age woman, it is necessary to have a high suspicion of ovarian ectopic pregnancy. Ovarian ectopic pregnancies are rare but are increasing in prevalence, and thus, swift diagnosis and treatment are prudent in preventing morbidity and mortality from delayed diagnosis.

## Conclusions

Ovarian ectopic pregnancies are rare, but evidence suggests that the prevalence is increasing. An ovarian ectopic pregnancy may be difficult to diagnose as the appearance on ultrasound may mimic that of a corpus luteal cyst, endometrioma, or another ovarian cyst. Laparoscopy or laparotomy with the histopathologic evaluation of the tissue is the gold standard for diagnosis. However, a high index of suspicion can be ascertained with the use of transvaginal ultrasound. The term "ring of fire" refers to a ring of vascularity that may be present around an ectopic pregnancy due to increased blood flow. However, the "ring of fire" may be seen around a normal corpus luteal cyst. Disturbance of a normal corpus luteal cyst may lead to miscarriage in a normal intrauterine pregnancy. A yolk sac or fetal pole with cardiac activity within or on the ovary offers the immediate diagnosis of ovarian ectopic pregnancy. If a yolk sac or fetal pole is not present, then the observer should search for a hyperechoic ring in the outer half to one-third of the ovary, which may depict the edge of the gestational sac. More research on the use of transvaginal ultrasound to identify ovarian ectopic pregnancies is needed, with dedicated attention to the sensitivity of a hyperechoic ring. Treatment modalities include both surgical and medical options, including methotrexate.
